# MYC Expression and Metabolic Redox Changes in Cancer Cells: A Synergy Able to Induce Chemoresistance

**DOI:** 10.1155/2019/7346492

**Published:** 2019-06-25

**Authors:** Barbara Marengo, Ombretta Garbarino, Andrea Speciale, Lorenzo Monteleone, Nicola Traverso, Cinzia Domenicotti

**Affiliations:** ^1^Department of Experimental Medicine, General Pathology Section, University of Genova, Italy; ^2^UOC Mutagenesis and Oncologic Prevention, Ospedale Policlinico San Martino, Genova, Italy

## Abstract

Chemoresistance is due to multiple factors including the induction of a metabolic adaptation of tumor cells. In fact, in these cells, stress conditions induced by therapies stimulate a metabolic reprogramming which involves the strengthening of various pathways such as glycolysis, glutaminolysis and the pentose phosphate pathway. This metabolic reprogramming is the result of a complex network of mechanisms that, through the activation of oncogenes (i.e., MYC, HIF1, and PI3K) or the downregulation of tumor suppressors (i.e., TP53), induces an increased expression of glucose and/or glutamine transporters and of glycolytic enzymes. Therefore, in order to overcome chemoresistance, it is necessary to develop combined therapies which are able to selectively and simultaneously act on the multiple molecular targets responsible for this adaptation. This review is focused on highlighting the role of MYC in modulating the epigenetic redox changes which are crucial in the acquisition of therapy resistance.

## 1. Cancer Metabolic Reprogramming

Metabolic reprogramming is an early event in the carcinogenic process, and it is involved in the development of malignancy and the acquisition of most cancer hallmarks [1]. The first metabolic phenotype observed in cancer cells was described by Otto Warburg, a German biochemist, as a shift from oxidative phosphorylation (OXPHOS) to aerobic glycolysis to generate lactate and ATP even in the presence of O_2_ (i.e., Warburg effect) [2]. Since the Warburg effect is also found in tumor cells with intact and functional mitochondria, it is reasonable to assume that it could represent a strategy adopted by cancer cells, not only to cope with the greater energy demands but also to reduce oxidative stress, preserving cells from oxidative death [3]. In this regard, reactive oxygen species (ROS), maintained at “physiological” levels, have been demonstrated to activate redox signaling pathways involved in cell proliferation and survival [4, 5].

Over the past decade, numerous studies have supported the hypothesis that the Warburg effect can be explained by the alterations in multiple signaling pathways resulting from mutations of oncogenes and tumor suppressor genes [6, 7]. Indeed, tumor metabolic reprogramming involves the activation of key metabolic pathways such as glycolysis, the pentose phosphate pathway, and glutaminolysis [8].

In this regard, it has been demonstrated that the glycolytic metabolic switch is due to a marked slowing down of the conversion of phosphoenolpyruvate into pyruvate, a reaction catalyzed by pyruvate kinase (PKM) [9]. Furthermore, in cancer cells, it has been observed that the presence of the low-activity dimeric form of PKM2 promotes the conversion of pyruvate to lactate [10] and that the increased levels of lactic acid detected in cancer patients are related to rapid tumor growth and high levels of metastases [11]. Moreover, considering that most chemotherapeutic agents are weak bases, the presence of lactic acid, generating acidity, induces the ionization of the drugs which, in their modified chemical structure, are not able to enter the tumor cells, thus facilitating the onset of chemoresistance [12, 13].

**Figure 1:**
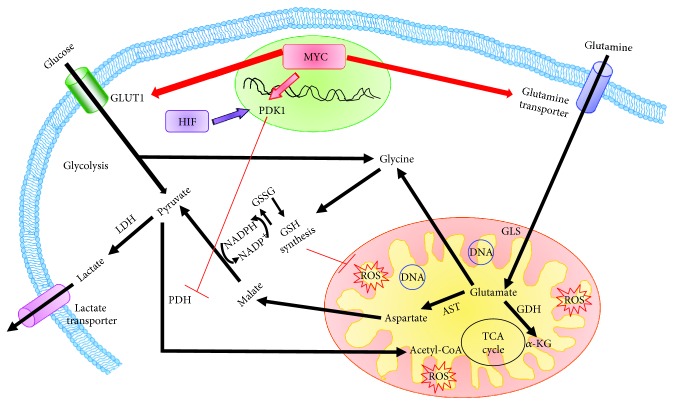
Role of MYC in the modulation of cancer metabolic reprogramming. MYC is involved in the modulation of both glycolysis and glutaminolysis. MYC, in order to carry out this double role, upregulates membrane transporters and enzymes involved in these metabolic processes (indicated in red). AST: glutamic-oxaloacetic transaminase; GDH: glutamate dehydrogenase; GLS: glutaminase; GLUT1: glucose transporter 1; GSH: reduced glutathione; GSSG: oxidized glutathione; *α*-KG: *α*-ketoglutarate; LDH: lactic dehydrogenase; PDH: pyruvate dehydrogenase; PDK1: pyruvate dehydrogenase kinase 1; ROS: reactive oxygen species; TCA: tricarboxylic acid.

PKM2, which makes cells less susceptible to oxidative stress and enhances NADPH production [14, 15], has been found to have a role in chemoresistance. In fact, a recent study showed that this kinase promotes gemcitabine resistance on one hand by inhibiting the transcriptional activation of p53 and the p38-mediated signaling pathway and on the other by increasing the expression of the antiapoptotic protein bcl-xl [16]. In addition, it has been reported that many cancer cells in order to satisfy their bioenergetic and metabolic needs depend on glutamine which is the main source of tricarboxylic acid (TCA) cycle precursors (Figure 1). For example, at the mitochondrial level, glutamine is converted to glutamate by glutaminase (GLS). In turn, glutamate can be converted to *α*-ketoglutarate (KG) by glutamate dehydrogenase (GDH) or transaminase, resulting in sustaining the TCA cycle. In addition, glutamate can serve as a precursor not only of nonessential amino acids such as aspartate, alanine, proline, and arginine but also of the most important intracellular antioxidant, glutathione (GSH), which is a tripeptide consisting of glutamate, cysteine, and glycine. In addition, malate, which is derived from glutamine, can be converted into pyruvate, leading to NADPH formation [17]. Therefore, the production of NADPH and GSH, derived from glutamine, allows cancer cells to reduce oxidative stress levels associated with mitochondrial respiration and rapid cell proliferation (Figure 1).

In this regard, our recent studies on human neuroblastoma (NB) cells [18], as well as other studies carried out on brain tumor samples [19] and ovarian cancer cells [20], have all demonstrated that the acquisition of chemoresistance is associated with high levels of GSH that enable cancer cells to counteract the prooxidant action of many chemotherapeutic agents [4, 21, 22].

It is noteworthy that the dependency of tumors on specific metabolic substrates, such as glucose or glutamine, is determined by alterations in oncogenes and oncosuppressor genes which are responsible for the tumor metabolic phenotype, while also supporting tumorigenesis. Among oncogenes, MYC has been found to have a pivotal role in the metabolic reprogramming of tumor cells by enhancing glucose uptake and glycolysis, lactate production and export, glutamine uptake and glutaminolysis, mitochondrial biogenesis, and oxidative phosphorylation [1].

## 2. Role of MYC in Cancer Metabolic Reprogramming and Adaptation to Therapy

MYC is a family of protooncogenes (i.e., c-MYC, L-MYC and N-MYC) which encode transcription factors that have roles in both normal and cancer cell physiologies. MYC requires dimerization with the protein MAX for DNA binding and for the assembly of transcriptional machinery. MAX can also interact with Mxd members which are transcriptional repressors and act in antagonism with MYC/MAX complexes. In addition, Mxd members can also bind to Mlx proteins that can interact with transcription activators of the Mondo family [23]. The MondoA/Mlx complex, located in the cytosol, translocates to the nucleus where, in response to an increase in extracellular glucose levels, it stimulates the expression of the thioredoxin-interacting protein (TXNIP) which suppresses the glucose uptake by limiting the expression of glucose transporters (GLUT) in the membrane [24, 25].

MYC is strongly involved in regulating cell metabolism and facilitates glycolysis by inducing the activation of genes encoding for glycolytic enzymes and GLUT (Figure 1) [26]. It is also able to promote mitochondrial biogenesis and function, thus increasing both oxygen consumption and ATP production [27–29].

Furthermore, it has been found that MYC upregulates the expression of glutamine transporters, facilitating glutaminolysis [30, 31], which is also stimulated by repressing microRNA-23a/b transcription leading to GLS1 overexpression [32]. As reported above, GLS converts glutamine to glutamate [32] which either enters the TCA cycle for the production of ATP or serves as a substrate for GSH synthesis [30]. In this regard, it has been reported that S6K1, a downstream effector of mTORC1, facilitates the translation of MYC, further contributing to the increase of GLS and GDH [33, 34]. In addition, it has been shown that mTORC1 expression, in response to stress conditions, is inhibited by FOXO transcription factors [35] and an increased expression of FOXO3a is able to antagonize the MYC binding to promoters, reducing the mitochondrial mass, oxygen consumption, and ROS production [36].

Regarding the enhancement of the mitochondrial function, it has been found that MYC can activate the PPAR*γ* coactivator-1*α* (PGC-1*α*) and the mitochondrial transcription factor A (TFAM), mediators of mitochondrial biogenesis and mitochondrial gene expression, respectively [28, 37]. Interestingly, although the role played by MYC/PGC-1*α* axis is controversial [38], several reports have demonstrated that PGC1-*α* is involved in chemoresistance [39] and the inhibition of the PGC-1*α* pathway has been found to activate glycolysis [40] and to sensitize melanoma to oxidative damage [41].

**Figure 2:**
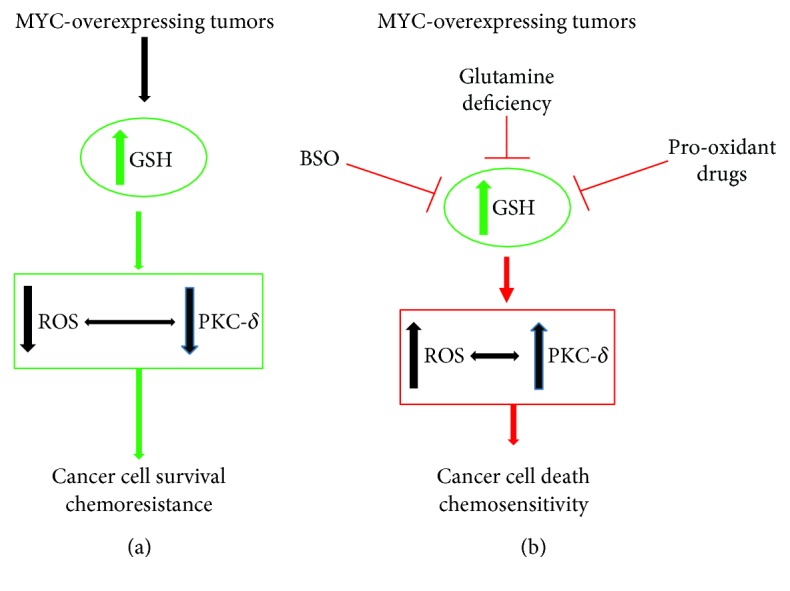
MYC overexpression and increase of glutathione levels in the acquisition of chemoresistance. (a) Chemoresistance of MYC-overexpressing tumors is associated with an enhancement of intracellular glutathione (GSH) levels. (b) In order to promote cell death, it is helpful to deplete GSH by using depleting agents such as buthionine sulfoximine (BSO) or prooxidant drugs. These strategies stimulate reactive oxygen species (ROS) production which modulate, and are modulated by, the proapoptotic protein kinase C-delta (PKC-*δ*).

Therefore, as reported above, the MYC-overexpressing tumors depend on glutamine [30, 31], and it has been demonstrated that glutamine depletion leads to the reduction of GSH levels and consequently triggers apoptosis. In fact, buthionine sulfoximine- (BSO-) induced depletion of GSH was able to induce apoptosis of N-MYC-amplified NB cells through a ROS-mediated activation of PKC*δ*-dependent pathways (Figure 2) [5, 42, 43]. Accordingly, PKC*δ* overexpression sensitized NB cells to the proapoptotic effects of BSO and of etoposide [18, 44–46].

Clinical studies carried out on NB patients have demonstrated that N-MYC amplification correlates to a reduction in the survival rate of those patients undergoing a multidrug therapy protocol consisting of etoposide, vincristine, carboplatin, adriamycin, and cyclophosphamide [47].

## 3. Molecular Mechanisms of MYC-Dependent Metabolic Changes

In N-MYC-amplified NB tumors, Akt has been found to be hyperactivated [48] and Akt activation has been demonstrated to be strongly involved in etoposide resistance [46, 49–51], as well as being related to the expression of CD133, a marker of staminality associated with the most aggressive cancer phenotype [52]. Accordingly, it has been shown that Akt inhibition sensitizes NB cells to the cytotoxic action of etoposide [53], doxorubicin, vincristine, and cisplatin [52]. In addition, under conditions of nutrient deficiency, the reduced activity of Akt decreases the amount of MDM2, the p53 endogenous inhibitor, resulting in an increase in p53 levels [54]. In fact, it has been found that the activation of p53 limits glycolysis and promotes OXPHOS in cancer cells while the loss of function of mutated p53 contributes to the development of the Warburg effect [55, 56]. Therefore, p53, in repressing PGC-1*α*, which is involved in mitochondrial biogenesis, and modulating other genes implicated in autophagy, in glucose metabolism, and also in the pentose-phosphate pathway [57–66], can play a role as a regulator of tumor cell metabolism and chemoresistance.

Interestingly, our recent studies have shown that chronic treatment of N-MYC-amplified NB cells with etoposide does not modify the homozygous p53 mutation (A161T), previously found in etoposide-sensitive NB cells, and therefore, in this context, p53 is responsible neither for OXPHOS activation nor for the metabolic adaptation of etoposide-resistant NB cells [67].

Moreover, several studies have demonstrated that the metabolic reprogramming might be the result of the “molecular interplay” between N-MYC and hypoxia-inducible factors (HIFs) [68]. HIF1 and HIF2 provide transcriptional homeostatic responses to limited oxygen levels in both physiological and pathological conditions. Although physiological HIF1 can inhibit the activity of normal MYC, the altered expression of the oncogenic MYC collaborates with HIF to confer the propensity to cancer cells to convert glucose to lactate, even in the presence of adequate O_2_ levels [69–72]. In fact, at normal MYC levels, it has been observed that HIF1*α* can compete for MAX, displacing MYC, while, at higher MYC levels, the formation of MYC-MAX heterodimers is maintained through mass action. Similar to MYC, HIF1 activates all genes involved in glycolysis, but unlike MYC, HIF1 actively inhibits mitochondrial respiration by promoting mitochondrial autophagy [73, 74] and preventing mitochondrial biogenesis [29]. In this context, it has been reported that HIF1 induces the expression of pyruvate dehydrogenase kinase (PDK1) which phosphorylates and inactivates pyruvate dehydrogenase, a mitochondrial enzyme catalyzing the conversion of pyruvate to acetyl CoA [75, 76]. Moreover, it has been found that MYC, when overexpressed in human tumors, cooperates with HIF1 to induce PDK1 and hexokinase 2 (HK2) expression, altering cellular metabolism in favor of glycolysis with an increased production of lactate [70, 75]. HIF1 and MYC independently activate GLUT1 and lactate dehydrogenase A (LDHA), resulting in an increased glucose influx and higher glycolytic rates [75].

Interestingly, HK2, which plays a key role for the Warburg effect in cancer, binds competitively to the voltage-dependent anion channel (VDAC), in the outer mitochondrial membrane, preventing its union with proapoptotic Bax and thereby avoiding apoptosis [77].

Apoptosis and senescence represent two tumor-suppressive mechanisms which can be modulated by MYC and RAS oncogenes. In fact, RAS inhibits MYC-induced apoptosis *via* PI3K activity and MYC suppresses RAS-induced senescence *via* CdK2, a cyclin-dependent kinase which phosphorylates MYC at Ser62 residue [78]. Accordingly, CdK2 inhibition has been shown to slow down the growth of MYCN-amplified neuroblastoma cells [79] and of other MYC-driven tumors [80].

Many chemotherapeutic drugs exert their cytotoxic effects on cancer cells by reactivating apoptosis and/or senescence [81]. In this context, it has been hypothesized that therapy-induced senescence (TIS) could be useful in the treatment of tumors with an impairment of the apoptotic pathways.

Interestingly, it is relevant to know that the presence of TIS cells can stimulate immunosurveillance and also induce chemoresistance [82, 83]. In fact, TIS cells have features of stemness that is regulated by the Wnt-dependent pathways [84–86] and undergo a metabolic reprogramming characterized by an increase in the glycolytic activity [2, 3] and an impairment of proteasome activity and autophagy [87]. In this context, the treatment of oncologic patients with anthracyclines and alkylating agents has been shown to induce cellular senescence and the secretion of cytokines, chemokines, growth factors, and proteases that can contribute to the side effects of chemotherapy [82, 88].

Recently, it has been reported that downregulation of p21, a cell cycle inhibitor, leads to MYC upregulation which represses the expression of CD47 receptor generating a subpopulation of cells that escape senescence [89]. However, further studies are necessary to determine if senescence is a general adaptive pathway to chemotherapy and if this response concerns only a specific subpopulation of cancer cells.

## 4. Inhibition of MYC Effectors as a Potential Strategy to Block Cancer Metabolic Reprogramming

Although MYC is considered the “most-wanted” target for anticancer therapy [90], the targeting of this oncogene has not yet obtained any positive outcomes. In fact, the inhibition of MYC can interfere with its physiological functions and therefore an alternative approach inhibiting MYC effectors could be more useful. More specifically, given that MYC drives the glucose and glutamine metabolism of cancer cells, the use of small molecules, able to inhibit enzymes involved in glycolysis and glutaminolysis, might be effective in slowing down tumor cell proliferation. Among them, several drugs targeting the MYC effectors are currently being tested in clinical practice [91–97] (Table 1).

Interestingly, a promising approach could be to indirectly modulate MYC through the “synthetic lethality” [90], and in this regard, the development of MK-3475 (pembrolizumab or keytruda) might offer new therapeutic opportunities. In fact, this latter compound is an inhibitor of the programmed death-1 (PD-1) protein and MYC modulates the expression of its ligand (PD-L1) [98], which, when overexpressed, stimulates glucose metabolism [99] by increasing GLUT1 expression [100]. MK-3475 has been, and is currently, the subject of over 900 clinical trials, and two of these have even reached Phase 4 (NCT03715205; NCT03134456). In addition, in Phase 3 studies, it should be noted that this compound per se is efficacious in treating recurrent or metastatic head-and-neck squamous cell carcinoma (NCT02252042) [101], advanced urothelial carcinoma (NCT02256436) [102], non-small-cell lung cancer (NCT01905657) [103], and melanoma (NCT02362594) [104].

## 5. Conclusions

Tumor metabolic reprogramming is a direct result of the reengineering of intracellular signaling pathways that are altered by activated oncogenes or downregulated oncosuppressors and by epigenetic changes, conferring a proliferative advantage to cancer cells.

Indeed, tumors may prefer either a glycolytic or an oxidative metabolism, depending on the activation of oncogenes or repression of oncosuppressors but also on the tumor microenvironment. Therefore, it is conceivable that in the tumor niche there is a strong “metabolic competition” due to high nutritional requirements and also an intense “molecular interplay” able to maintain an efficient metabolism. The balance between these factors could paradoxically guarantee the development and the survival of cancer even under therapy-induced stress conditions. Consequently, therapies that block glucose metabolism might be more effective towards tumors with high glycolytic rates, while they might develop therapy resistance in tumors whose metabolism depends on oxidative phosphorylation [105].

Therefore, anticancer therapy must take into account that most chemotherapeutic drugs are prooxidant agents and are able to induce a metabolic reprogramming that alters the redox homeostasis of cancer cells activating signaling pathways responsible for cell survival.

Considering the crucial role of MYC in driving the metabolic reprogramming of cancer which has been shown to be strictly related to drug resistance, several studies have been carried out in order to focus MYC-dependent metabolic pathways. Even though the efforts are multiple, to date the applicability of MYC inhibitors is still a utopia. However, the use of small molecules, able to inhibit MYC-related enzymes involved in glycolysis and glutaminolysis, might result effective in slowing down tumor cell proliferation and counteracting chemoresistance.

However, the characterization of the metabolic reprogramming of tumors and its connection with oncogenic signaling is a promising strategy to identify novel molecular approaches in anticancer treatment.

## Figures and Tables

**Table 1:** Drugs targeting glucose or glutamine metabolism currently used in clinical trials.

Drug	Target	Effect on MYC	Cancer type	Phase trials	NCT
Silibyn	GLUT	Reduction [91]	Prostate cancer	II	00487721

Gossypol	Lactate dehydrogenase (LDH)	Reduction [92]	Small-cell lung carcinoma	II	00773955
			Prostate cancer	II	00666666
			Esophageal/gastroesophageal cancer	I/II	00561197
			Glioblastoma	I	00390403
				II	00540722

Dichloroacetate	Pyruvate dehydrogenase (PDH)	Reduction [93]	Breast cancer and non-small-cell lung carcinoma	II	01029925
			Head and neck cancer	I	01163487
			Brain cancer	II	00540176

Deoxyglucose	Hexokinase II	Reduction [94]	Prostate cancer	I/II	00633087
			Lung cancer and breast cancer	I	00096707

Apigenin	Pyruvate kinase M (PKM)	Reduction [95]	Breast cancer	—	03139227

Diclofenac	GLUT1 and LDH	Reduction [96]	Basal cell carcinoma	II	01358045

CB-839	Glutaminase1 (GLS1)	Reduction [97]	Leukemia	I	02071927
			Colorectal cancer	I/II	02861300
			Hematological tumors	I	02071888
			Melanoma	I/II	02771626
			Triple negative breast cancer and solid tumors	I	02071862
